# A pivotal role of vacuolar H^+^-ATPase in regulation of lipid production in *Phaeodactylum tricornutum*

**DOI:** 10.1038/srep31319

**Published:** 2016-08-08

**Authors:** Huiying Zhang, Rensen Zeng, Daoyi Chen, Jian Liu

**Affiliations:** 1 College of Life Sciences, Fujian Agriculture and Forestry University, Fuzhou 350002, China; 2 Center for Molecular Cell and Systems biology, College of Life Sciences, Fujian Agriculture and Forestry University, Fuzhou 350002, China; 3 Division of Ocean Science and Technology, Graduate School at Shenzhen, Tsinghua University, Shenzhen 518055, China

## Abstract

Microalgal lipids have been considered as a promising source for biodiesel production. Alkaline pH can induce neutral lipid accumulation in microalgae cells. However, whether and how proton pumps, especially vacuolar H^+^-ATPase (V-ATPase), function in these processes is not well known. In this study, we treated *Phaeodactylum tricornutum* with V-ATPase specific inhibitor bafilomycin A1 (BFA1) to determine its role in lipid production. Firstly, V-ATPase activity was increased in the latter phase of microalgae growth. BFA1 treatment decreased the cell density and lipid contents. Further analysis showed that BFA1 treatment reduced the number and size of oil bodies. GC-MS analysis showed that lipid components were not affected by BFA1 treatment. Intracellular pH was decreased and nitrogen depletion was delayed after BFA1 treatment. RNA-Seq analysis showed that expression of genes involved in calcium signaling, sulfur metabolism, cell cycle, glycolysis, pentose phosphate pathway, porphyrin, chlorophyll metabolism and lipid catabolic metabolism were upregulated, while expression of genes involved in ion transmembrane transport, ubiquitin mediated proteolysis, SNARE interactions in vesicular transport, fatty acid biosynthesis were downregulated under BFA1 treatment. Our findings provided insights into the molecular mechanisms underlying lipid accumulation and the key genes involved in lipid metabolism in *Phaeodactylum tricornutum* in response to BFA1.

Biodiesel is a new type of green bioenergy, which has attracted considerable attention as a potential alternative to fossil fuels^1^. Microalgal cells can store carbon and energy in the form of lipids, which can be easily converted to biodiesel, and some diatoms accumulate neutral lipids; thus, microalgae, particularly diatoms, have been considered as a promising biodiesel feedstock^2^. Diatoms are a diverse group of eukaryotic unicellular microalgae, which contribute up to 40% of marine productivity^3^. *Phaeodactylum tricornutum* (*P*. *tricornutum*) is a widely utilized model system in studying the ecology, physiology, biochemistry, and molecular biology of diatoms; the genome of this diatom has been sequenced and is available at the Ensembl Genome databases^4^. Furthermore, *P*. *tricornutum* has attracted increasing attention as a raw material for biofuel production because it rapidly grows to high cell densities, has a short biomass doubling time, and accumulates triacylglycerols (TAGs) in the late exponential phase; storage lipids constitute at least 20–30% of the dry cell weight of this diatom under standard culture conditions^5^.

Most algaes can produce both starch and lipids as energy reserves, with ratios that differ depending on growth conditions. In the most common situation, starch is the primary energy compound. Reserve lipids usually serve as a secondary energy source and an electron sink when their production is more economical for the cell than production of starch^6^. Lipids can be divided into two groups: polar lipids, which are components of cell membranes and organelles, and non-polar or neutral lipids, which serve as the energy reserves^7^. Under unfavorable growth conditions, many algaes alter their lipid biosynthetic pathways to induce the formation and accumulation of neutral lipids, mainly in the form of TAGs^8^. Unlike the glycerolipids found in membranes, TAGs play no structural roles and primarily serve as a storage form of carbon and energy^9^. However, evidence suggests that the TAG biosynthetic pathway plays an active role not only in carbon and energy storage but also in response to environmental stress conditions^8^. In vascular plants, individual classes of lipid are synthesized and localized in a specific cell, tissue, or organ; in algae, different types of lipid are synthesized in a single algal cell. After being synthesized, TAGs are deposited in densely packed oil bodies in the cytoplasm of the algal cell^6^.

Many environment factors regulate the production of microalgae lipid, such as nutrient deprivation, temperature, light density^10^,^11^,^12^. Besides, microalgaes are also sensitive to changes in pH, which is crucial to maintain high growth rates. Buffers or inorganic acids are usually used to control pH, which is simple and can also help pressure a pure culture^13^. Guckert and Cooksy demonstrated that cellular TAG accumulation can be induced in a single species of *Chlorella* by growing it at a pH higher than normal^14^. This process was explored further and was proven applicable to other microalgal genera, such as *Scenedesmus*, *Coelastrella* and *P*. *tricornutum*^15^,^16^. A recent study has demonstrated that lipid amounts are comparably equal or higher under pH stress conditions than under standard nutrient-deprivation treatments in five isolates of freshwater green algae^17^. Analysis of transcripts suggested the metabolic pathway and repurposing of phospholipids were altered under the pH stress^16^. However, how pH stress influences intracellular pH and the effect of intracellular pH in lipid production remain unclear.

There are three different membrane proton pumps capable of generating pH gradients in plants^18^. The plasma membrane H^+^-ATPase is an important proton pump in the plant cell membrane, which is composed of a single polypeptide. It is a key molecular player that determines and controls plant nutrient uptake, cell elongation, leaf movements and intracellular pH homeostasis^19^. H^+^-pyrophosphatases (H^+^-PPase) is highly hydrophobic single-subunit protein that also generates proton gradients across endomembrane compartments using the energy of the phosphoanhydride bond of pyrophosphate molecules^20^. Vacuolar H^+^-ATPase (V-ATPase) is a highly conserved multisubunit enzyme complex, which consists of 14 different subunits and two subcomplexes. The peripheral V1 domain is composed of eight distinct subunits (A_3_, B_3_, C_1_, D_1_, E_2_, F_1_, G_2_, and H_1–2_) and functions in ATP hydrolysis, whereas the integral V0 domain consists of six different subunits (a_1_, d_1_, e_n_, c_4–5_, c′_1_, and c′′_1_) and is responsible for proton translocation^21^. V-ATPase is localized in various subcellular organelles in plants, such as endoplasmic reticulum, Golgi apparatus, and vacuoles^22^. The V-ATPase has been proved to be involved in embryonic development, cell expansion and the acidification of vacuole and other intracellular trafficking compartments^23^,^24^. Additionally, V-ATPase subunits had also been revealed in microalgae. Kitade *et al*. reported the nucleotide sequence of a gene encoding the c subunit of the V-ATPase from a marine red alga, *Porphyra yezoensis*^25^. An immunological observation indicated that the level of subunit B of V-ATPase increased under high concentration of CO_2_^26^. However, information on the role of these proton pumps, especially V-ATPase in lipid regulation is lacking.

Bafilomycin A1 (BFA1) is a membrane-permeant compound belonging to a class of macrolide antibiotics known to specifically inhibit the V-ATPase^27^. In *Brassica napus*, treatment with 50 nM BFA1 decreased V-ATPase activity by 36.6%^28^. Additionally, previous study had also revealed that treatment with BFA1 could induce a small alkalinization of the vacuole, and a significant acidification of the cytoplasm^29^. Therefore, we used BFA1 as V-ATPase inhibitors to determine the role of intracellular pH in microalgal lipid production. In this study, by using RNA-seq approach, we demonstrated that V-ATPase plays an important role in the regulation of lipid production, which is associated with energy metabolism, ion transport and cell cycle control.

## Result and Discussion

### An increased V-ATPase activity in the culture of *P*. *tricornutum*

Each microalgae has its own optimal culture conditions for pH value. For example, the optimal pH for the growth of *Pseudochlorella* sp. YKT1, *Chlorella pyrenoidosa*, *P*. *tricornutum* is pH 3.0–5.0, pH 5.7–6.5, pH 7.8–8.5, respectively^16^,^30^,^31^. In fact, due to uptake of inorganic carbon by algae, pH can rise significantly in algal culture. Our data showed that the medium pH was increased with the prolongation of culture time in *P*. *tricornutum*. The medium pH peaked after 3 d of growth, slowly decreased, and finally stabilized at pH 9.5 in 5 d after inoculation (Figure S1). We speculated that the fluctuation of medium pH might be due to the alteration of intracellular pH.

**Figure 1:**
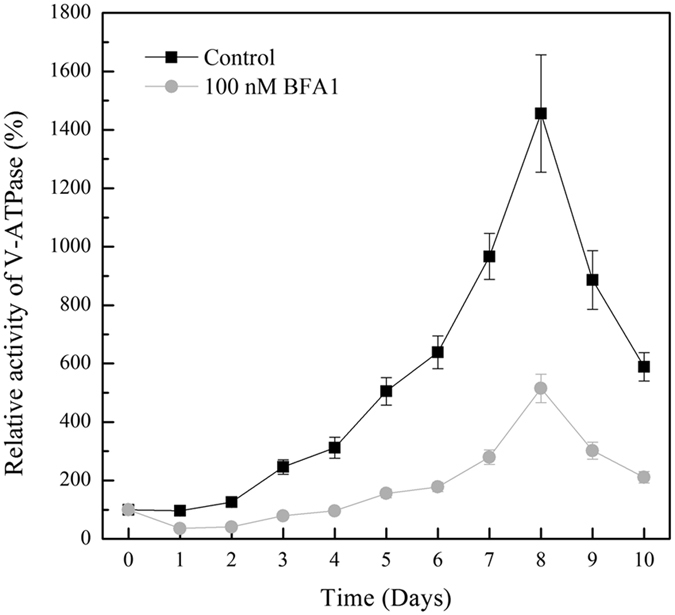
V-ATPase activity during *Phaeodactylum tricornutum* culture. During the culture of *P*. *tricornutum* (10 days), the activity of V-ATPase under 100 nM BFA1 treatment or not were calculated as the difference measured in the absence or presence of 100 nM Con A in the required time. Values shown represent mean ± SE (n = 3).

Under alkaline conditions whereby the extracellular pH is higher than intracellular pH, the cell must rely on active transport of HCO_3_^−^ and not on passive flux of CO_2_ for inorganic carbon accumulation^32^. V-ATPase is a major proton pump in the proton homeostasis of eukaryotic cells. Therefore, to explore the key role of V-ATPase on the culture of *P*. *tricornutum*, V-ATPase special inhibitor Bafilomycin A1 (BFA1) was used. The efficiency and toxicity of BFA1 were determined (Figure S2). Treatment with 100 nM BFA1 had no significant influence on the cell viability. To study the role of V-ATPase in different culture phase, BFA1 was added in the initial culture or 6 days after culture. The activity and proton pumping of V-ATPase were determined after 8 days culture. It was shown that both of them were significantly decreased under the treatment of 100 nM BFA1. Additionally, The V-ATPase activity was also determined during the whole culture period. The V-ATPase activity was increased at the third day, peaked at 8 days after culture, and then decreased (Fig. 1). However, the V-ATPase activity under 100 nM BFA1 was decreased by nearly 66% all through the growing period (Fig. 1). The increased activity of proton pumps might promote the cytoplasmic alkalization in the later phase of *P*. *tricornutum* growth.

### Changes in intracellular pH under BFA1 treatment

The acetoxymethyl ester of 2′, 7′-bis-(2-carboxyethyl)-5-(and-6)-carboxyfluorescein (BCECF) is a nonfluorescent molecule that easily penetrates cells^33^. Upon reaching the cytoplasm, the AM groups were cleaved by the action of nonspecific esterases, yielding the highly fluorescent molecule BCECF. After excitation at 480–550 nm, the emission intensity of BCECF at 525–535 nm pH-dependently increased. To determine intracellular pH, the ratio determined between a pH-dependent emission intensity and a pH-independent emission intensity is usually determined^34^.

**Figure 2:**
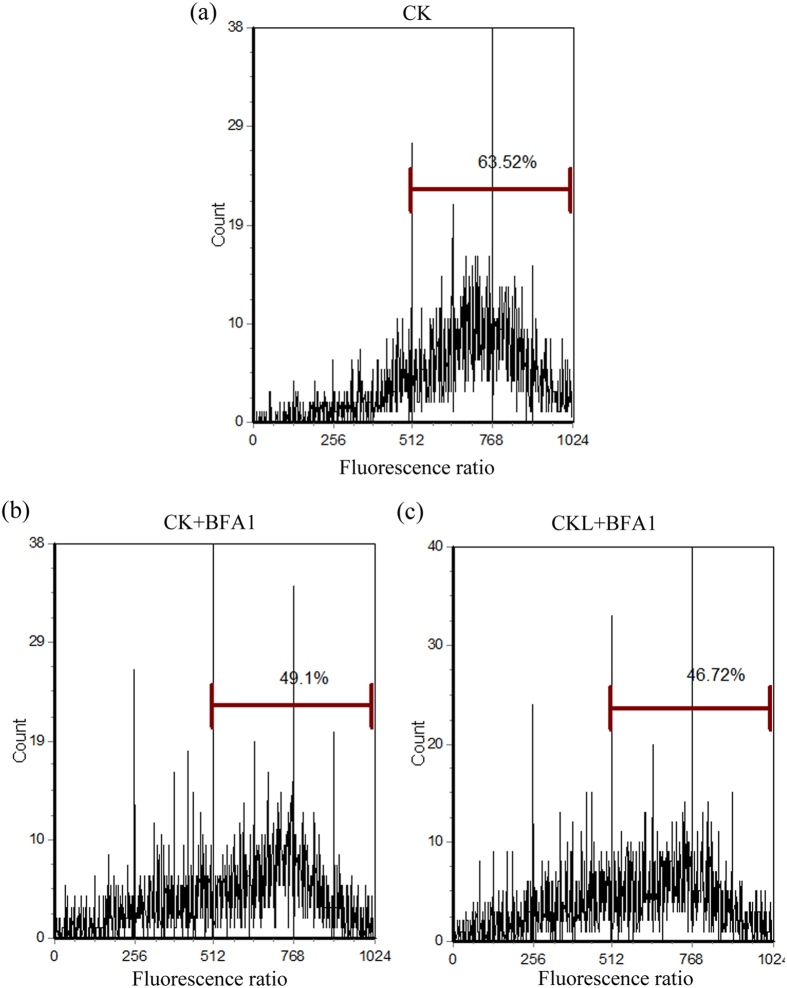
Intracellular pH of *Phaeodactylum tricornutum* treated with BFA1. After 8 d of culture, cells were rinsed once with PBS buffer and then resuspended in the same solution. BCECF-AM was added to achieve a final concentration of 2 μL mL^−1^. Cells were incubated at 37 °C for 30 min to allow cleavage of AM ester. Aliquots of 10^6^ cells were removed, cells were centrifuged, and the supernatants were discarded. Pellets were resuspended in 1 mL of PBS. BCECF excitation was provided by the 488 nm line of an argon laser. When used with an Epics Elite cytometer, power as low as 15–20 mW was adequate for excitation. The resulting fluorescence was separated into high- and low-wavelength components by a 550 nm dichroic filter. These components were further narrowed by passing through 640 and 525 nm band pass filters. The ratio of 525/640 nm fluorescence was measured as reflected by intracellular pH alteration in control treatment (**a**), BFA1 treatment in the initial culture (**b**) and BFA1 treatment in 6 days after culture (**c**). Control (CK), no treatment; CK + BAF1, treatment with 100 nM BFA1 in the initial culture; CKL + BFA1, treatment with 100 nM BFA1 in 6 days after culture.

The calibration curve is obtained by plotting the mean fluorescence ratio of samples measured in high [K^+^] buffer and nigericin against pH (Figure S3). The marker bar H was set to indicate cells with BCECF efflux, which was measured by counting cells in the H region of the plot. After the 8-day culture, the percentage of control cells exhibiting a high BCECF fluorescence was 63.52%, whereas that of 100 nM BFA1 treatment in the initial culture and 6 days after culture was 49.1% and 46.72%, respectively (Fig. 2). In contrast to the calibration curve, the intracellular pH in control cells was 8.0–8.5, whereas that of BFA1 treatment in the initial culture and 6 days after culture was 6.5–7.0 and 6.0–6.5, respectively. This indicates an important role of V-ATPase in regulation of intracellular pH in *P*. *tricornutum*.

### Growth rates under BFA1 treatment

**Figure 3:**
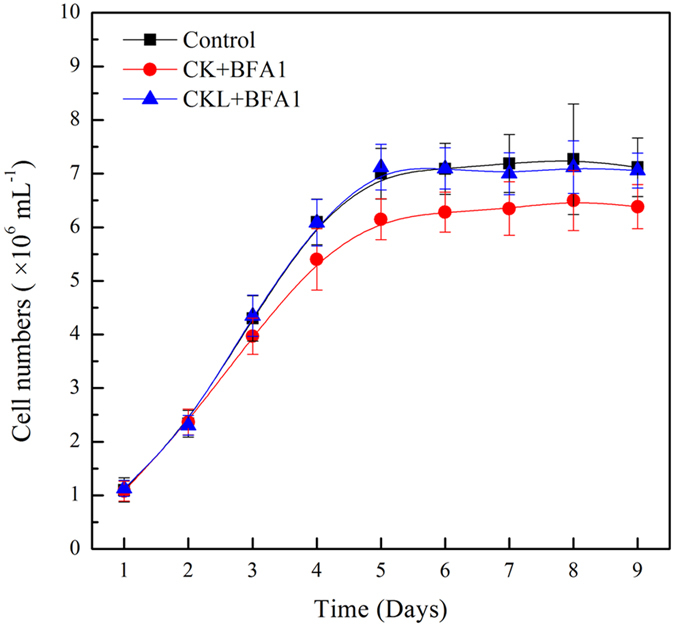
Cell numbers during the culture of *Phaeodactylum tricornutum* with BFA1 treatment. Cell numbers of *P*. *tricornutum* were counted daily using an Olympus microscope and a Brightline Hemocytometer. Control (CK), no treatment; CK + BAF1, treatment with 100 nM BFA1 in the initial culture; CKL + BFA1, treatment with 100 nM BFA1 in 6 days after culture. Each bar represents three replications.

Maximum algal growth occurs around neutral pH, although optimum pH is the initial culture pH at which an alga is adapted to grow^32^. To examine the role of V-ATPase in the culture of *P*. *tricornutum*, 100 nM BFA1 was added in the initial culture or 6 days after culture. The cell density over the culture cycle was determined. As shown in Fig. 3, without treatment, the cell density peaked after 6 d in culture, and then reached a plateau. And treatment with 100 nM BFA1 in 6 days after culture didn’t influence the cell growth. While under the treatment of 100 nM BFA1 in the initial culture, cell density was decreased over the culture cycle. Changing pH in media may limit algal growth^35^.

### TAG accumulation under BFA1 treatment

Previous study had revealed that alkaline pH stress results in triglyceride accumulation in *Chlorella CHLOR1*^14^. However, the effect of pH stress on the lipid production in microalgae is varied. A higher alkaline pH appears not to enhance the starvation-induced increase in lipid contents in *Neochloris oleoabundans*^36^, suggesting a high specificity of *Neochloris oleoabundans* cell machinery towards TAG production. Additionally, the way of pH treatment also influenced the lipid production. Incrementally adjusted pH over the course of growth triggered lipid accumulation comparable to constant pH stress treatments, yet biomass accumulation was equivalent to unstressed growth in five isolates of freshwater green algae^17^. Therefore, lipid amounts were comparably equal or better for pH stress treatments than for standard nutrient-deprivation treatments in some microalgaes.

**Figure 4:**
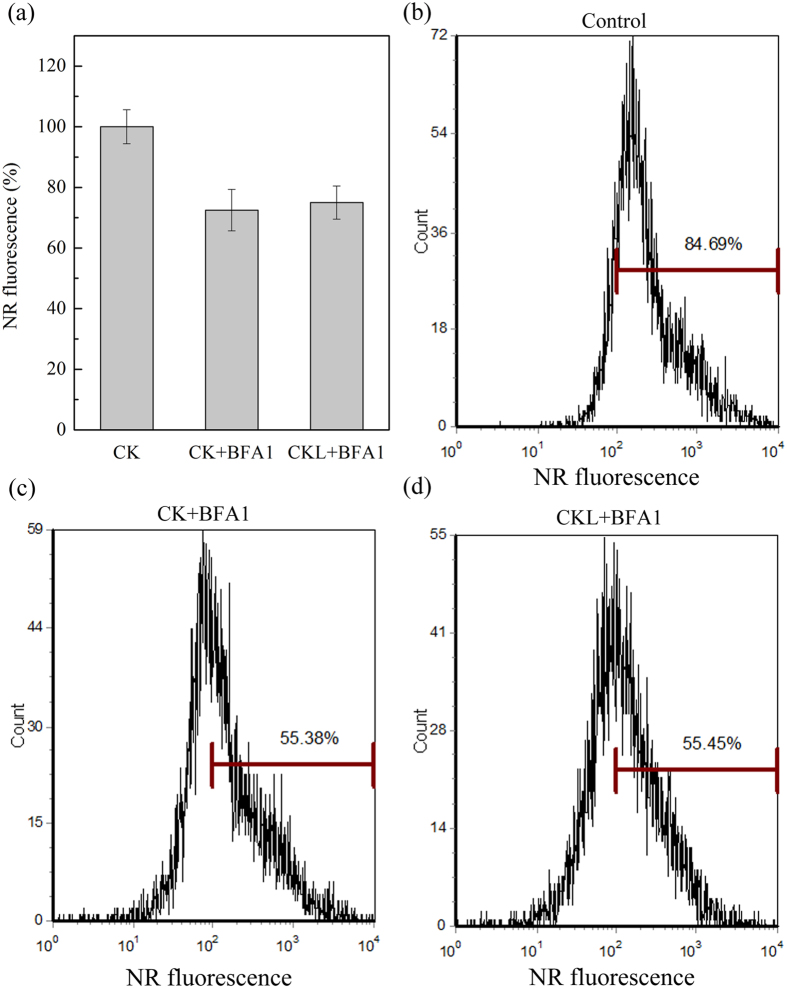
TAGs accumulation during the culture of *Phaeodactylum tricornutum*. (**a)** Nile Red (2 μL of a 0.1 mg mL^−1^ acetone solution) was added to 200 μL of *P*. *tricornutum* culture medium. Nile red fluorescence was determined using a microtiter plate reader. Each bar represents three replications. (**b, c, d**) Nile red (5 μL of a 0.1 mg mL^−1^ acetone solution) was added to 100 μL of *P*. *tricornutum* culture medium. Nile red fluorescence was determined using a flow cytometer equipped with a xenon ion excitation lamp (excitation wavelength, 488 nm; emission maximum, 595 nm). The marker bar H was set to indicate cells with high nile red efflux, which was measured by counting cells in the H region of the plot. The percentage of control cells exhibits a high nile red fluorescence. Control (CK), no treatment; CK + BAF1, treatment with 100 nM BFA1 in the initial culture; CKL + BFA1, treatment with 100 nM BFA1 in 6 days after culture.

TAG accumulation under 100 nM BFA1 treatment was monitored by nile red fluorescence and confirmed by gas chromatography. Nile red staining of neutral lipids is a well-characterized method for the rapid screening of oil accumulation in algal cells. The lipid contents of the cultures were assessed using nile red and a microtiter plate reader with a 480 nm excitation wavelength and a 592 nm emission wavelength. The content of neutral lipids was both decreased under 100 nM BFA1 treatment compared with that in control (Fig. 4). Furthermore, the fluorescence intensity per cell was also determined through flow cytometry. The marker bar H was set to indicate cells with high nile red efflux, which was measured by counting cells in the H region of the plot. Similar results were found in treatment with 100 nM BFA1. The percentage of control cells exhibiting a high nile red fluorescence was 84.69%, while approximately 55.38% and 55.45% of microalgal cells exhibited a high nile red fluorescence, in culture with BFA1 treated in the initial culture and 6 days after culture, respectively (Fig. 4). These results suggest that treatment with BFA1 can reduce the accumulation of neutral lipids in *P*. *tricornutum*.

### Fatty acid composition under BFA1 treatment

The former study revealed that relative fatty acid contents of the TAGs pool are independent from pH applied at either cultivation stage in *Scenedesmus obliquus*^12^. However, structural lipids are reduced when pH increases to high levels^37^. Hence, regulating the pH during algae cultivation might refine the lipid composition in the harvested algal biomass.

GC–MS analysis revealed that C20:5 (eicosapentaenoic) fatty acid was the most abundant in *P*. *tricornutum*, followed by C16:1 (palmitoleic), C16:0 (palmitic) and C18:1 (oleic) fatty acid (Table 1). Both of the treatment with 100 nM BFA1 decreased the content of fatty acid. These results further suggested that treatment with BFA1 can suppress the lipid production in *P*. *tricornutum*. However, no significant difference was found in fatty acid composition between BFA1-treated and control cells, which suggested that BFA1 treatment didn’t alter membrane lipid fatty acid profiles.

### Observation of oil bodies

**Figure 5:**
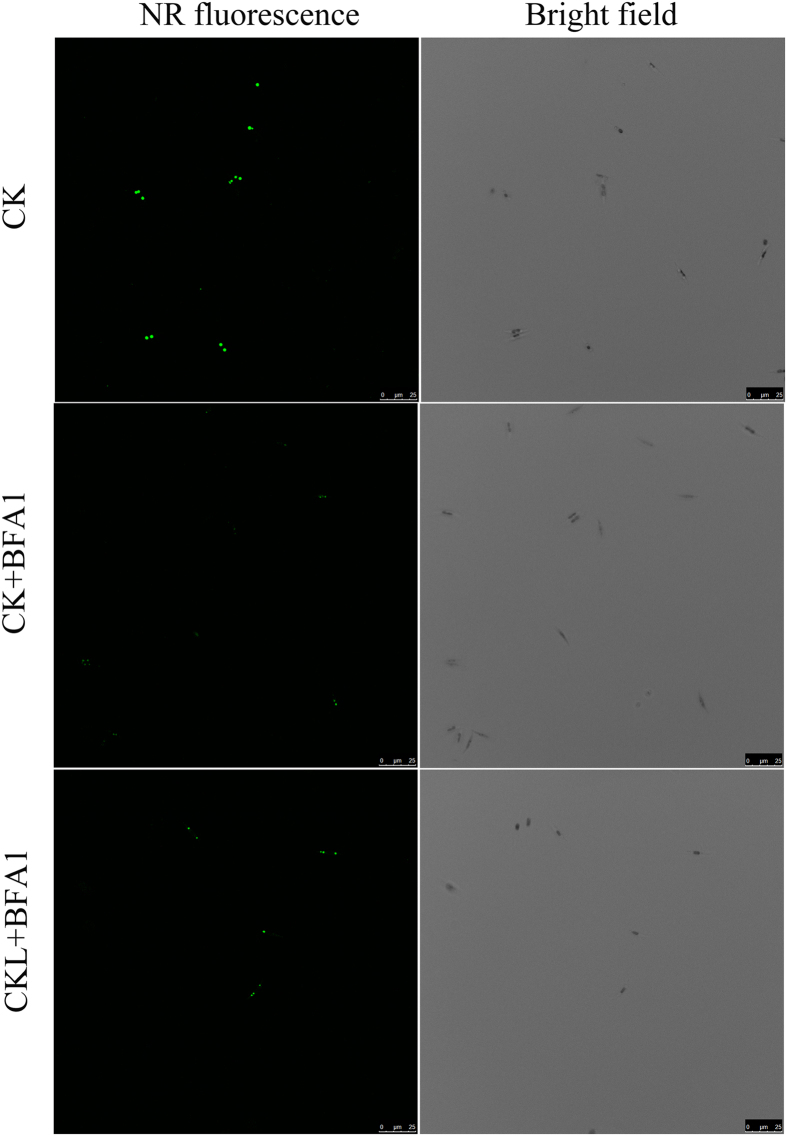
Oil bodies observed by confocal microscopy. Cells sampled after 8 d of culture were stained with nile Red. Mixtures were thoroughly mixed, applied to a glass slide, covered with a coverslip after 10 min, and then observed under an LSM 510 META laser-scanning confocal microscope (Zeiss), with 543 nm excitation wavelength and 570–610 nm emission wavelength. Images were acquired randomly from at least 20 cells per sample, and typical images are presented here. Control (CK), no treatment; CK + BAF1, treatment with 100 nM BFA1 in the initial culture; CKL + BFA1, treatment with 100 nM BFA1 in 6 days after culture. Bar = 25 μm.

Neutral fatty acids are deposited in densely packed oil bodies in the cytoplasm of the algal cell^6^. To visually assess the relative quantity and spatial conformation of oil accumulation, neutral lipid droplets was also studied by laser scanning confocal microscope and transmission electron microscope. The size and number of oil bodies in the sampled cells were observed after 8 d of culture. Decreased NR fluorescence was observed under BFA1 treatment than that in control (Fig. 5). Our previous studies revealed that 2-[N-cyclohexylamino]-ethane-sulfonic acid (CHES) treatment promoted the lipid accumulation in *P*. *tricornutum*. To further elucidate the role of V-ATPase in pH-induced lipid accumulation, 100 nM BFA1 was added in CHES-treated microalgae cells. The oil body was also examined by confocal microscopy. The numbers and sizes of oil bodies were both decreased in the combination of BFA1 and CHES treatment compared with that in only CHES treatment (Figure S4), which further indicated that a key role of V-ATPase in lipid production.

**Figure 6:**
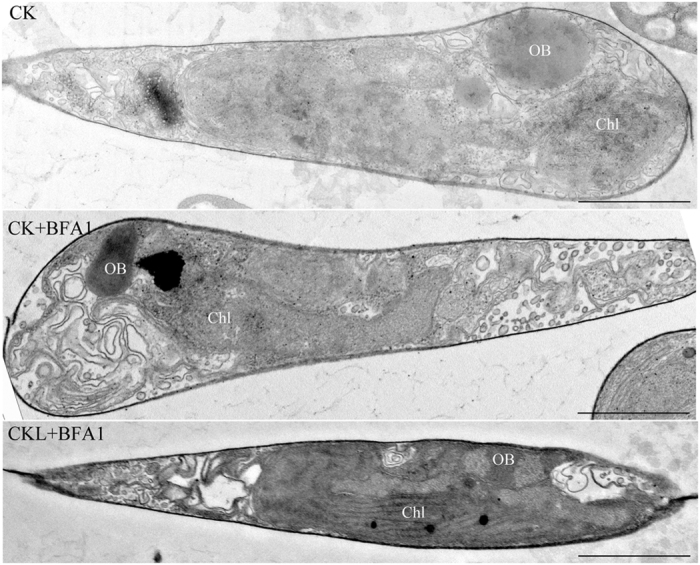
Effects of BFA1 treatment on *Phaeodactylum tricornutum* ultrastructure. Transmission electron micrographs showing subcellular structures of BFA1 treatment and control *P*. *tricornutum* cells. OB, oil body; Chl, chloroplast; Control (CK), no treatment; CK + BAF1, treatment with 100 nM BFA1 in the initial culture; CKL + BFA1, treatment with 100 nM BFA1 in 6 days after culture. Bar = 1 μm.

Additionally, the sizes of these oil bodies were also determined by transmission electron microscope. Smaller oil bodies were found under BFA1 treatment compared with that in control (Fig. 6). Therefore, the total volume of oil bodies was lower in the BFA1-treated cultures than in the control cultures, which agrees with the decrease in neutral lipid content (Fig. 4). Meanwhile, intracellular membranes basically remained the same (Fig. 6), which was consistent with the result of cell viability and fatty acid composition determination.

The most important function of oil bodies is to serve as a source of energy and carbon in case the supply from photosynthesis is not sufficient, or during recovery in replenished mineral medium following nutrient starvation^38^. However, the role of oil bodies in algae is not limited to their storage function but plays an important role in adaptation to environmental conditions^39^. V-ATPase had widely involved in stress response, such as salt and osmotic stress^40^, MeJA-induced leaf senescence^41^. However, the mechanism of V-ATPase-regulated lipid production is still unknown. To unravel the molecular mechanism of them, a comparative transcriptional analysis was taken.

### Global transcript differential expression

The V-ATPase activity was peaked at 8 days after culture. Therefore, we monitored the differences in gene expressions after the 8-day culture to investigate the molecular mechanisms underlying lipid accumulation in *P*. *tricornutum* under BFA1 treatment using RNA-Seq (Initial treatment and 6 days after culture). In brief, 521 and 212 genes were upregulated with an FDR cutoff of 0.1%, 588 and 226 genes were downregulated under the treatment with 100 nM BFA1 in the initial culture or 6 days after culture, respectively (Figures S5 and S6). This transcriptomes result suggested that V-ATPase inhibitor treatment caused a wide re-programming of regulation. And the effect of BFA1 on the transcriptional regulation was varied in accompany with treatment time. The transcriptomes results were validated through quantitative PCR (qPCR) analysis of eight selected genes over the treatment, including β-actin as an endogenous control, to test the differential expression (Table 2). The result of RNA-Seq measurement was consistent with that of qPCR, confirming their robustness. For example, under the treatment with 100 nM BFA1 in the initial culture, qPCR analysis and RNA-Seq measurement results revealed that Phatr3_J29488, which encodes a type of fatty acid desaturase (FADS), was upregulated by 3.5526 (log_2_)-fold and 4.3848 (log_2_)-fold, respectively. And that treated in 6 days after culture was upregulated by 4.5023 (log_2_)-fold and 4.6750 (log_2_)-fold, respectively.

The genes encoding all known enzymes in the *P*. *tricornutum* genome were mapped in KEGG pathways, together with log_2_-fold differences in RNA expression between control and BFA1 treatments. Gene ontology (GO) classification of the *P*. *tricornutum* transcripts with Blast2GO program under BFA1 treatment was shown in Figure S7. Genes involved in calcium signaling, sulfur metabolism and cell cycle, glycolysis/gluconeogenesis, pentose phosphate pathway, porphyrin and chlorophyll metabolism, and desaturase were upregulated. Genes involved in most of ion transmembrane transporter, ubiquitin mediated proteolysis, SNARE interactions in vesicular transport, fatty acid biosynthesis were downregulated.

### Ion transport

**Figure 7:**
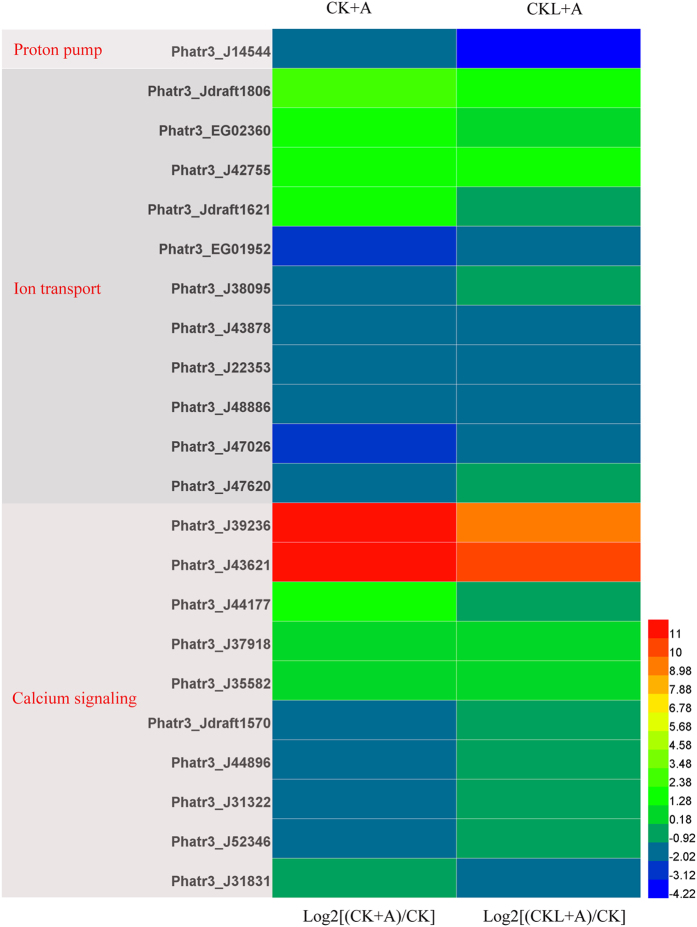
Transcript levels of genes encoding components involved in ion channel and calcium signaling under BFA1 treatment. Hierarchical clustering of transcriptional fold changes from three technical replicates of duplicate cultures (n = 2), relative to transcript levels in control. CK, no treatment; CK + A, treatment with 100 nM BFA1 in the initial culture; CKL + A, treatment with 100 nM BFA1 in 6 days after culture.

We noticed that gene encoding proton pumps was down-regulated under BFA1 treatment. BFA1 treatment in 6 days after culture decreased the gene Phatr3_J14544 encoding one subunit of V0 domain in V-ATPase by 18.60-fold, while that under BFA1 treatment in the initial culture only decreased by 2.73-fold (Fig. 7). Chemiosmotic circuits of plant cells are driven by proton (H^+^) gradients that mediate secondary active transport of compounds across plasma and endosomal membranes^42^. Our data (Fig. 7) showed that genes encoding inorganic anion exchangers and metal ion transmembrane transporter were up-regulated under BFA1 treatment. And voltage-gated ion channel, especially chloride channel, solute/proton antiporter and cation trasmembrane transporter were down-regulated in treatment with BFA1. The alteration of ion transport might influence the related enzyme activity, further regulating the metabolism process. Additionally, it is interesting that calmodulin-dependent protein kinase activity and calcium ion binding were both enhanced under the treatment with BFA1. Gene Phatr3_J39236 involved in calmodulin binding increased by 4958.32-fold with treatment of BFA1 in the initial culture, and that under BFA1 treatment in 6 days after culture increased by 900.81-fold. The exact role of calcium signaling in V-ATPase-regulated lipid production should be investigated in the future.

### Protein degration and transport

**Figure 8:**
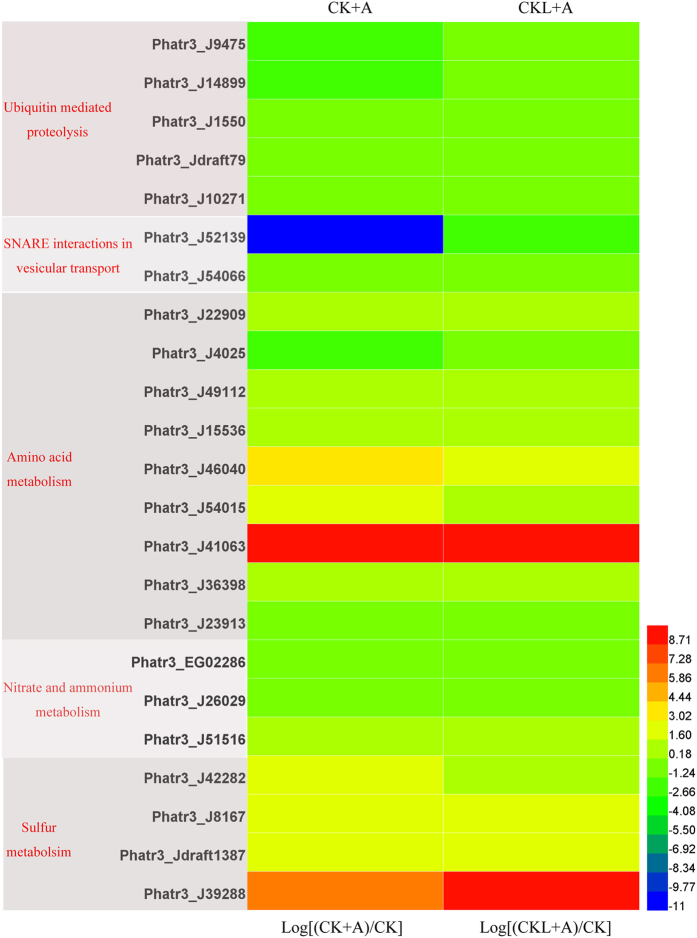
Transcript levels of genes encoding components involved in protein degradation and transport, nitrogen and sulfur metabolism under BFA1 treatment. Hierarchical clustering of transcriptional fold changes from three technical replicates of duplicate cultures (n = 2), relative to transcript levels in control. CK, no treatment; CK + A, treatment with 100 nM BFA1 in the initial culture; CKL + A, treatment with 100 nM BFA1 in 6 days after culture.

Previous study had revealed that the accumulation of lipids was a consequence of remodeling of intermediate metabolism. Specifically, approximately one-half of the cellular proteins were cannibalized^43^. One important proteolytic pathway involves the small protein ubiquitin (Ub) and the 26S proteasome, a 2-MDa protease complex^44^. We found that treatment with BFA1 significantly downregualted the ubiquitin mediated proteolysis. Genes Phatr3_J9475, Phatr3_J14899 involved in proteolytic pathway were decreased by 3.25-fold, 2.58-fold treated with BFA1 in the initial culture (Fig. 8; Table S1). The inhibitory effect of BFA1 on proteolytic pathway could lead to the delay of remodeling of intermediate metabolism. Additionally, the nitrogen was scavenged by urea and glutamine synthetase/glutamine 2-oxoglutarate aminotransferase pathways and redirected to the de novo synthesis of nitrogen assimilation machinery on nitrogen stress^43^. Our results revealed that genes involved in urea cycle had no significant alterations under the treatment with BFA1.

V-ATPase-driven proton pumping and organellar acidification is essential for vesicular trafficking along both the exocytotic and endocytic pathways of eukaryotic cells^21^. Our results found that SNARE interactions in vesicular transport were severely suppressed by the treatment with 100 nM BFA1 in both the initial time and late exponential phase. For example, gene Phatr3_J52139 was downregulated by 2330.17-fold and 3.27-fold treated with 100 nM BFA1 in the initial culture and 6 days after culture, respectively (Fig. 8; Table S1). SNAREs seem to mediate membrane fusion in all of the trafficking steps of the secretory pathway^45^. As we known, Oil bodies (OBs) consist of a hydrophobic central core of neutral lipids, such as TAGs, surrounded by a monolayer of amphipathic phospholipids, glycolipids, and/or sterols, with a series of proteins bound to the surface of the OB^46^. Therefore, addition of BFA1 might suppress the formation of OBs membrane.

### Nitrogen metabolism

Most algae can produce both starch and lipids as energy reserves. The ratio between starch and fatty acid synthesis correlated strongly to the biomass nitrogen content^47^. For all tested conditions, nitrogen limitation was a prerequisite for lipid accumulation^16^. TAG accumulation per cell, monitored by nile red fluorescence, correlates with pH at the time of nitrate depletion^15^.

**Figure 9:**
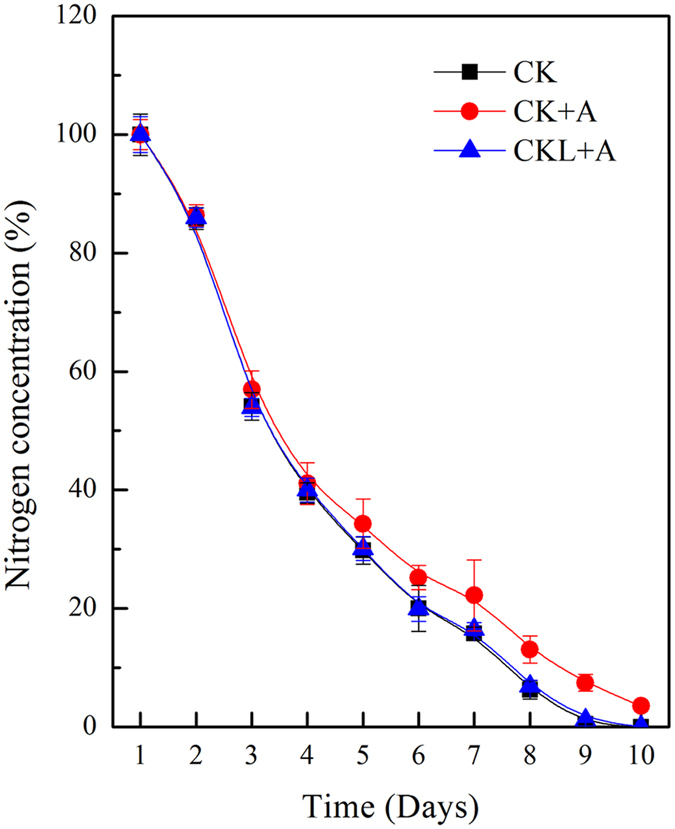
Nitrogen concentrations in the culture of *Phaeodactylum tricornutum* under BFA1 treatment. Nitrogen content was determined using the colorimetric method (Cleverchem200, DeChem-Tech). Nitrogen concentration was represented in the ratio nutrient concentration to initial concentration. Each bar represents three replications. CK, no treatment; CK + A, treatment with 100 nM BFA1 in the initial culture; CKL + A, treatment with 100 nM BFA1 in 6 days after culture.

Compared with control treatment, most of genes involved in amino acid metabolism were up-regulated (Fig. 8). Gene Phatr3_J41063 involved in glycine, serine and threonine metabolism was increased by 787.59-fold under the treatment with BFA1 in the initial culture, and that increased by 1040.13-fold treated with BFA1 in 6 days after culture; Gene Phatr3_J46040 involved in cysteine and methionine metabolism was increased by 9.87-fold treated with BFA1 in the initial culture, that increased by 6.13-fold with BFA1 treatment in 6 days after culture. Nevertheless, gene Phatr3_J4025 involved in lysine biosynthesis was reduced by 4.31-fold treated with BFA1 in the initial culture. The nitrogen sources taken up by higher plants are nitrate or ammonium as inorganic nitrogen sources^46^. Treated with BFA1 in the initial culture, our data showed that genes Phatr3_EG02286 and Phatr3_J26029 involved in nitrate metabolism were reduced by 2.28-fold and 2.09-fold, respectively. And gene Phatr3_J51516 involved in ammonium transport was increased by 2.06-fold. To further investigate the influence and interaction of pH and nitrogen concentration on lipid production, exogenous nitrate was examined daily to determine nutrient availability. The nitrogen contents were calibrated (Figure S8). Nitrogen depletion was delayed with BFA1 treatment in the initial culture, while no significant difference of nitrogen depletion was found in control and BFA1 treatment in 6 days after culture (Fig. 9). In Arabidopsis, V-ATPase activity was acquired for efficient nutrient storage^33^. However, the previous study had revealed that alkaline pH-induced TAG accumulation was independent of medium nitrogen or carbon levels^14^. Therefore, the exact role of V-ATPase on nitrogen metabolism should be further investigation in the future.

### Sulfur metabolism and cell cycle

Under the deprivation of sulfur, macromolecular syntheses (RNA, protein) were arrested during the second cell cycle concomitantly with blocked nuclear and cellular division in *S*. *Quadricauda*^48^. DNA replication, nuclear and cellular division use the reserves to meet their carbon and energy requirements. It is proposed that only cells unable to complete a cell cycle will accumulate lipid^49^. Under the treatment with BFA1 in the initial culture, our data was shown that gene Phatr3_J42282 containing sulfate adenylyltransferase activity, gene Phatr3_J8167 encoding thioredox m, gene Phatr3_Jdraft1387 containing sulfotransferase activity were increased by 6.25-fold, 6.0-fold, 5.92-fold, respectively. Treated with BFA1 in 6 days after culture, gene Phatr3_J39288 containing sulfotransferase activity was increased by 1117.97-fold. Additionally, we noticed that gene Phatr3_EG02598 encoding Rad51 which functioned in double-stranded-break repair in S phase was increased by 15.09-fold under the treatment with BFA1 in the initial culture. However, we found the cell density was slightly decreased by the treatment with BFA1 in the initial time (Fig. 3). It seems that other factors induced by V-ATPase influence the process of cell cycle.

### Photosynthesis

**Figure 10:**
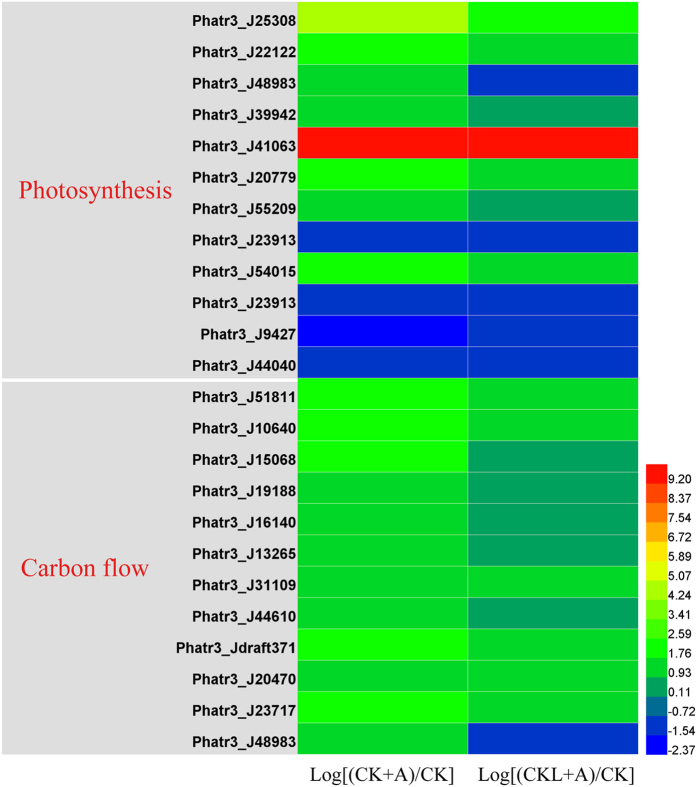
Transcript levels of genes encoding components involved in glycolysis, transporter, TCA cycle under BFA1 treatment. Hierarchical clustering of transcriptional fold changes from three technical replicates of duplicate cultures (n = 2), relative to transcript levels in control. CK, no treatment; CK + A, treatment with 100 nM BFA1 in the initial culture; CKL + A, treatment with 100 nM BFA1 in 6 days after culture.

As shown in Fig. 10 and Table S1, levels of most transcripts encoding proteins associated with porphyrin and chlorophyll metabolism, photosynthesis increased under BFA1 treatment, implicating that photosynthesis could be enhanced. Notably, transcript levels of ferredoxin—NADP^+^ reductase (Phatr3_J23717), which catalyzes the last electron transfer, from photosystem I to NADPH during photosynthesis, increased by 4.59-fold and 2.19-fold under the treatment of BFA1 in the first time and 6 days after culture, respectively. Furthermore, genes related to porphyrin and chlorophyll metabolism were also upregulated. Genes Phatr3_J51811 encoding hydroxymethylbilane synthase, Phatr3_J10640 encoding coproporphyrinogen oxidase, Phatr3_J19188 encoding uroporphyrinogen decarboxylase, Phatr3_J31109 encoding protoporphyrinogen oxidase were increased by 3.84-fold, 3.73-fold, 3.07-fold and 2.69-fold in treatment with BFA1 in the initial time, respectively. And gene Phatr3_J10640 encoding coproporphyrinogen oxidase and Phatr3_J51811 encoding hydroxymethylbilane synthase were upregulated by 2.99-fold and 2.0-fold treated with BFA1 in the late exponential phase.

### Carbon flow

Inorganic carbon is a fundamental component for microalgal lipid biosynthesis. The observed carbon partitioning is caused by competition between fatty acid and starch synthesis for a common carbon pre-cursor^50^,^38^. Both starch and lipid metabolism start with a common initial pool of molecules consisting of three carbons, such as 3-phosphoglycerate (3PG) and glyceraldehyde 3-phosphate (GAP)^51^. Our data found that most genes involved in glycolysis were upregulated under BFA1 treatment (Fig. 10). Genes Phatr3_J22122 and Phatr3_J25308 encoding glyceraldehyde 3-phosphate dehydrogenase were increased by 3.05-fold and 20.12-fold treated with BFA1 in the initial culture. Moreover, gene Phatr3_J20779 encoding fructose-6-phosphate-aldolase was increased by 4.82-fold and gene Phatr3_J9427 encoding Udp-n-acetylglucosamine transferase subunit was reduced by 5.17-fold under the treatment with BFA1 in the initial culture. Glycolysis and pentose phosphate pathways are the major contributors of pyruvate production in vascular plant, pyruvate is then converted to acetyl-CoA by the pyruvate dehydrogenase complex (PDHC) for de novo fatty acid biosynthesis in the plastid^52^.

### Fatty acid metabolism

**Figure 11:**
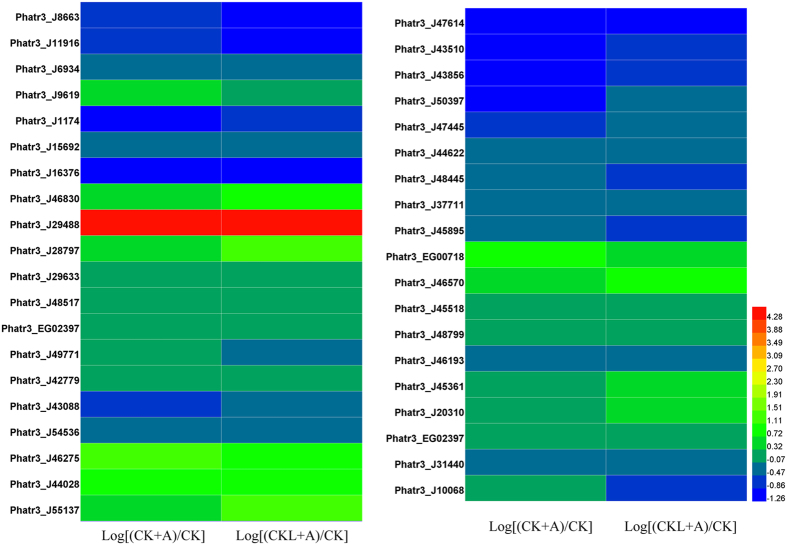
Transcript levels of genes encoding components involved in lipid metabolism under BFA1 treatment. Hierarchical clustering of transcriptional fold changes from three technical replicates of duplicate cultures (n = 2), relative to transcript levels in control. CK, no treatment; CK + A, treatment with 100 nM BFA1 in the initial culture; CKL + A, treatment with 100 nM BFA1 in 6 days after culture.

Most of the genes involved in fatty acid biosynthetic process were down-regulated and some of genes involved in lipid catabolic process were up-regulated under the treatment with BFA1 (Fig. 11). Gene Phatr3_J31440 encoding acyl carrier protein was reduced by 1.32-fold treated with BFA1 in the initial culture, and reduced by 1.24-fold treated with BFA1 in 6 days after culture. Gene Phatr3_J10068 encoding enoyl-ACP reductase was reduced by 1.57-fold treated with BFA1 in 6 days after culture. Additionally, genes Phatr3_J8663, Phatr3_J11916 and Phatr3_6934 involved in phospholipid biosynthetic process were reduced by 1.52-fold, 1.71-fold and 1.39-fold in treatment with BFA1 in the initial culture, and reduced by 2.04-fold, 2.20-fold and 1.38-fold treated with BFA1 in 6 days after culture. Gene Phatr3_J1174 involved in glycolipid biosynthetic process was reduced by 1.30-fold and 1.46-fold treated with BFA1 in the initial culture and 6 days after culture, respectively. Gene Phatr3_J16376 involved in elongation of fatty acid was reduced by 1.92-fold in treatment with BFA1 in the initial culture, and reduced by 1.87-fold treated with BFA1 in 6 days after culture. Furthermore, genes Phatr3_J46830, Phatr3_J29488, Phatr3_J28797 encoding delta 5, 6, 9 fatty acid desaturase were increased by 1.40-fold, 19-fold and 1.30-fold in treatment with BFA1 in the initial culture, and increased by 1.90-fold, 25.54-fold and 2.40-fold treated with BFA1 in 6 days after culture, respectively. The alteration of these genes involved in fatty acid bisynthetic process might lead to the decrease in lipid content, especially the neutral lipids levels.

Some genes involved in lipid catabolic metabolism were upregulated. Genes Phatr3_J20310 and Phatr3_EG02397 containing acyl-CoA dehydrogenase activity were increased by 1.09-fold and 1.13-fold in treatment with BFA1 in the initial culture, and increased by 1.32-fold and 1.11-fold treated with BFA1 in 6 days after culture, respectively. Previous study had revealed that increased cAMP levels promoted lipid breakdown^53^. Our data showed that genes Phatr3_J43510 and Phatr3_J48445 containing ephosphoric diester hydrolase activity were reduced by 2.15-fold and 1.37-fold treated with BFA1 in the initial culture, and reduced by 1.44-fold and 1.45-fold under the treatment with BFA1 in 6 days after culture, respectively. These might result in the enhancement of cAMP signaling. Integrating these signaling, BFA1 treatment might lead to the decrease of lipid content.

## Conclusions

This study provides a detailed physiological and molecular-level foundation for improved understanding of diatom nutrient cycling and contributes to a metabolic blueprint for controlling lipid accumulation in diatoms. Treatment with V-ATPase inhibitor BFA1 in the initial culture suppressed the microalgae growth while it had no significant effect on the growth treated with BFA1 in 6 days after culture. Both BFA1 treatment in the initial culture and 6 days after culture inhibited the lipid production. However, the lipid composition had no obviously changed. In combination with the biochemical approach, our results uncovered the pivotal metabolic pathways in the diatom under BFA1 treatment and provided a global view of biosynthetic metabolic fluxes. The details are as follows. The direct role is gene involved in fatty acid biosynthesis such as acyl carrier protein and enoyl-ACP reductase was downregulated, lipid catabolic metabolism such as gene encoding protein containing acyl-CoA dehydrogenase activity was upregulated. Then, sulfur metabolism and cell cycle were accelerated, and glycolysis/gluconeogenesis, pentose phosphate pathway, chlorophyll metabolism were upregulated. The carbon flow might shunt to starch production other than lipid production. Additionally, ubiquitin mediated proteolysis and SNARE interactions in vesicular transport were downregulated, which might result in lack of membrane of oil body. Finally the deficiency of the gene encoding ion transmembrane transporter might influence the enzyme activity involved in metabolism pathways. Therefore, these findings provide insights into several mechanisms that lead to lipid accumulation under BFA1 treatment, which reflect to the function of V-ATPase in microalgal lipid production.

In summary, V-ATPase functions a key role in diatom cultures and lipid accumulation. We also provide strategies that can be applied to manipulate the biosynthetic pathways of microalgae and generate cultures with high levels of lipids that may be suitable for biodiesel production.

## Materials and Methods

### Algal cultures

The marine diatom *P*. *tricornutum* was provided by Prof. Hongye Li of the Algal Collection Center of Jinan University (Strain No. FACHB-863). Wild-type cells were grown as batch cultures in flasks containing f/2-Si medium. Cultures were routinely cultivated in an artificial climate incubator under constant irradiance (150 μmol photon∙m^−2^ s^−1^), temperature (21 ± 0.5 °C), and 12/12-h light/dark cycles^54^.

CHES treatment was applied as follow. The medium used for the experiments was modified using 25 mM CHES buffer, and the initial pH was set to 9.5. For BFA1 treatment, 100 nM BFA1 was treated in the initial culture or in 6 days after culture.

### Cell density determination

Cell numbers were counted using an optical hemocytometer with a minimum of 400 cells according to the previous study^16^.

### Neutral lipid content analysis

Nile red is an excellent vital stain for the detection of intracellular lipid droplets by flow cytoflourometry and fluorescence microscopy^55^. Stock solutions of nile red (30 μL of a 0.1 mg mL^−1^ acetone solution) was added to 3 mL portions of cell cultures in triplicate. The resulting suspensions were mixed by rapid inversion and incubated in darkness for a minimum of 5 min at room temperature. For folw cytofluorometry, nile red fluorescence was also determined with a xenon ion excitation lamp (excitation wavelength, 488 nm; emission maximum, 595 nm). For a microplate reader, nile red fluorescence was quantified with 480/592 nm excitation/emission filters. Unstained samples were used for background medium and cellular autofluorescence correction.

### Fatty acid composition analysis

To determine the profile of fatty acid, total lipids were extracted from three independent biological replicates and lipid samples were trans-esterified in accordance with a previous method^56^,^57^. The resulting fatty acid methyl esters were analyzed by GC–MS with a 30 m × 0.25 mm × 0.25 μm DB-5 quartz capillary column. The injector temperature was 280 °C with an oven temperature gradient of 60 to 160 °C at 10 °C min^−1^ after a 1-min hold time at 60 °C, then with an oven temperature gradient of 160 to 250 °C at 2.5 °C min^−1^ after a 1-min hold. 1 μL samples were injected in splitless mode. The mass spectrum transmission line temperature was 200 °C. The relative contents of detected fatty acids were calculated using the normalization method.

### Observation of oil bodies

All microscopic observations were performed using a Zeiss LSM 510 META confocal laser-scanning microscope. For the observation of oil bodies, *P*. *tricornutum* cells were stained with nile red for 10 min. Nile red signals were visualized with excitation at 543 nm and emission at 570–610 nm using a band-pass filter^54^. Typical images are presented here.

### Determination of fluorescein diacetate (FDA) fluorescence

*P*. *tricornutum* was cultured with 100 nM BFA1 treatment for 2, 4, 6 days, then the cell was stained with 50 μM FDA. The FDA fluorescence was determined by a microtiter plate reader with excitation at 488 nm and emission at 530 nm^58^.

### Ultrastructural analysis by transmission electron microscopy

*P*. *tricornutum* cells were fixed and embedded as described previously^54^. Ultrathin sections were stained with uranyl acetate and lead citrate. The stained sections were examined under a transmission electron microscope (JEM-1200EX).

### Intracellular pH determination

After culturing for 8 d, cells were rinsed once with PBS buffer and then resuspended in the same solution. BCECF-AM was added to achieve a final concentration of 2 μL mL^−1^. Cells were incubated at 37 °C for 30 min to allow cleavage of AM ester. Aliquots of 10^6^ cells were removed, cells were centrifuged, and the supernatants were discarded. Pellets were resuspended in 1 mL of PBS. For calibration samples, the pellet was resuspended in high [K^+^] with different pH (6.0, 6.5, 7.0, 7.5, 8.0, and 8.5). Nigericin at 1 μL mL^−1^ was added 2–3 min prior to pH measurement^34^.

Excitation of BCECF was provided by the 488 nm line of an argon laser. The resulting fluorescence was separated into high- and low-wavelength components by a 550 nm dichroic filter. These components were further narrowed by passing through 640 and 525 nm band pass filters. The ratio of 525/640 nm fluorescence was measured.

### Determination of nitrogen content

Nitrogen content was determined using the colorimetric method (Cleverchem200, DeChem-Tech) according to the previous study^59^.

### Determination of V-ATPase activity and proton pumping ability

The membrane protein was extracted by the kit (BeiBo, BB-3170). Ten micrograms of microsomal membrane protein was applied to analyze the V-ATPase activity with 10 μg BSA as a negative control. The V-ATPase activity was calculated as the difference measured with or without 100 nM Concanamycin A, and the absorbance of phosphor was examined according to the previous method^40^. Proton pump activity of V-ATPase was assayed by the quenching of ACMA (9-amino-6chloro-2-methoxyacridine) fluorescence. The reaction buffer contained 250 mM sorbitol, 50 mM KCl, 3 mM ATP, 50 μM NaVO_4_, 1 mM NaN_3_, 2 μM ACMA and 10 mM MES-Tris (pH 7.5). MgSO_4_ (3 mM) was used to initiate the reaction. The fluorescence quenching was measured using a 410 nm excitation light and an emission wavelength of 480 nm by an auto microplate reader^41^.

### Validation using Real-time PCR

To further validate the RNA-Seq results, RNA extracted from the same culture for RNA-Seq was subjected to the PrimeScript RT reagent kit with gDNA Eraser (Takara) for cDNA synthesis. qPCR was performed using standard methods as previously described^40^. β-actin was used as a housekeeping marker. Primer pairs used for qPCR analysis are listed in Table 2.

### Transcriptome sampling and sequencing

After 8-day culture, RNA was isolated from the control, consistent BFA1-treated and BFA1-treated in 6 days after culture strains^52^. For mRNA-Seq, the extracted mRNA was enriched using oligo (dT) magnetic beads and were fragmented into short sequences (about 200 bp). The fragmented mRNA was converted into double-stranded cDNA by priming with random hexamer and purified with a QIAquick PCR extraction kit, then subjected to end repair and single nucleotide A (adenine) addition^54^. Finally, sequencing adaptors were ligated to the fragments. The required fragments were purified through agarose gel electrophoresis, enriched through PCR amplification, and then sequenced using a HiSeq™ 2000 (Illumina) instrument, with default quality parameters, at GENE DENOVO (Guangzhou, China).

The *P*. *tricornutum* reference genome was obtained from http://genome.jgi-psf.org/Phatr3/Phatr3.download.ftp.html. The *P*. *tricornutum* genome sequence is composed of “finished chromosomes” (Phatr3) and “unmapped sequence” (Phatr3_bd), which were annotated separately. Both portals were included in this study to allow a complete analysis of the genome. We aligned our RNA short reads (22977642 in total) under normal conditions to the reference genome using SOAPaligner/soap2 (GENE DENOVO, Guangzhou, China), which resulted in 14777041 mapped reads (64.31%). Under BFA1 treatment in the initial culture or 6 days after culture, we aligned 21132010 reads and 21980870 reads, with 12644252 (59.83%) and 14384856 (62.91%) mapping to the genome, respectively. Any gene with one read or less was discarded as nonconfident. The gene expression level is calculated by using RPKM method (Reads per kilobase per million mapped reads), and the formula is shown as follows:


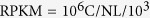


Given RPKM (A) to be the expression of gene A, C to be number of reads that uniquely aligned to gene A, N to be total number of reads that uniquely aligned to all genes, and L to be number of bases on gene A. The RPKM method is able to eliminate the influence of different gene length and sequencing discrepancy on the calculation of gene expression. Therefore, the calculated gene expression can be directly used for comparing the difference of gene expression among samples. And log_2_-transformed for each gene defined in the Phatrdraft_database to estimate the fold change upon BFA1 treatment^54^.

## Additional Information

**How to cite this article**: Zhang, H. *et al*. A pivotal role of vacuolar H^+^-ATPase in regulation of lipid production in *Phaeodactylum tricornutum*. *Sci. Rep*. **6**, 31319; doi: 10.1038/srep31319 (2016).

## Supplementary Material

Supplementary Information

Supplementary Data

## Figures and Tables

**Table 1:** Fatty acid composition of *P*. *tricornutum* following BFA1 deprivation.

Fatty acids	CK (ppm)	CK + BFA1 (ppm)	CKL + BFA1 (ppm)
C14:0 (myristic)	180.59	134.95	134.23
C16:0 (palmitic)	268.81	223.43	239.52
C18:0 (stearic)	46.52	56.43	61.47
C24:0 (Methyl tetracosanoate)	1.20	1.35	1.33
Sum SFA	497.12	416.16	436.55
C16:1 (palmitoleic)	887.47	758.62	829.63
C18:1 (oleic)	256.29	117.41	200.02
Sum MUFA	1143.76	876.03	1029.65
C18:2 (linoleic)	39.39	34.15	65.63
C18:3 (linolenic)	2.89	0.71	1.42
C20:5 (eicosapentaenoic)	2116.69	1666.45	1720.39
C22:6 (docosahexaenoic)	5.09	4.11	3.83
Sum PUFA	2164.06	1705.42	1791.27
Lipid content	3804.94	2997.61	3257.47

^*^CK, no treatment; CK + BFA1, treatment with 100 nM BFA1 in the initial culture; CKL + BFA1, treatment with 100 nM BFA1 in 6 days after culture; SFA, Saturated fatty acids; MUFA, Monounsaturated fatty acids; PUFA, Polyunsaturated fatty acids.

**Table 2:** qPCR of differentially expressed genes following BFA1 treatment.

Locus tag	Forward (5′-3′)	Reverse (5′-3′)	CK + A			CKL + A		
			qPCR FC Log_2_(E/CK)	RNA-Seq		qPCR FC Log_2_(E/CK)	RNA-Seq	
				FC Log_2_(E/CK)	FDR		FC Log_2_(E/CK)	FDR
32747	GCCCTCAACGGCAAACTCAC	TGTCCAGACGGCAAGTCAAGT	7.0355	7.369	0.2748	8.369	8.1856	0.1119
25308	CGCCCTCAACGGTAAACTCAC	GCACAGATGGTGGCGTATGG	3.8562	4.33	0.0003	2.15	2.4462	0.0065
46040	ACAGTCCAGCAGCGTAGTTAGG	TGTTGTCCCCTATTACATAAAGCAT	3.0452	3.3024	0.0007	2.5374	2.6161	0.0169
27877	ACGACACCACCGACAAGACCT	GCAACTGTACCGGCAACAATC	−0.0348	−0.0440	0.1070	−0.5153	−0.6080	8.18E-124
29488	GATTTCTGGAAGCTCCAAGTCAC	GCTGGGGAATAAGTGGTGGTC	3.5526	4.3848	0.0015	4.5023	4.6750	0.0019
41423	GTGGTTTGAAGCCCTTGTGC	CGCCCTGTTCGTAGTGCTTA	−0.3896	−0.6160	3.23E-71	−0.5230	−0.4213	1.92E-38
41063	ACGAATCCATCACCGCAAAT	GCTTTCGTAGATGCGGTTGAC	8.1442	9.6213	0.0046	9.0854	10.0225	0.0008
21988	TGCTTCATCTTTCGGCTGCTA	AGGCTCTATTTCGTCCTTGCG	−0.1863	−0.2098	6.94E-225	−0.0856	−0.0395	9.41E-10
32747	GCCCTCAACGGCAAACTCAC	TGTCCAGACGGCAAGTCAAGT	5.7186	7.3694	0.2749	7.5568	8.1856	0.1119

^*^CK, no treatment; CK + A, treatment with 100 nM BFA1 in the initial culture; CKL + A, treatment with 100 nM BFA1 in 6 days after culture.
