# Supplementary material for: A pivotal role of vacuolar H+-ATPase in regulation of lipid production in Phaeodactylum tricornutum
Source: Sci Rep. 2016 Aug 8;6:31319. doi: 10.1038/srep31319 (PMC4976316; doi:10.1038/srep31319)
Supplement: Supplementary Data [file srep31319-s2.doc]

A pivotal role of vacuolar H^+^-ATPase in regulation of lipid production in *Phaeodactylum tricornutum*

Huiying Zhang^1, 2, 3^, Rensen Zeng^1^, Daoyi Chen^3^*, Jian Liu^1^*

**Supplementary data**

**Table S1. Fold changes in the expression of some genes encoding enzymes involved in various metabolisms after BFA1 treatment.** Comparative RNAseq was used to investigate the molecular mechanisms underlying lipid accumulation in *P. tricornutum* under BFA1 treatment in the initial culture or in 6 days after culture. The gene expression level is calculated by using RPKM method. And log2-transformed for each gene defined in the Phatrdraft_database to estimate the fold change upon BAF1 treatment. Detail method was shown in method. And genes correlated with hierarchical clustering were shown here.

**
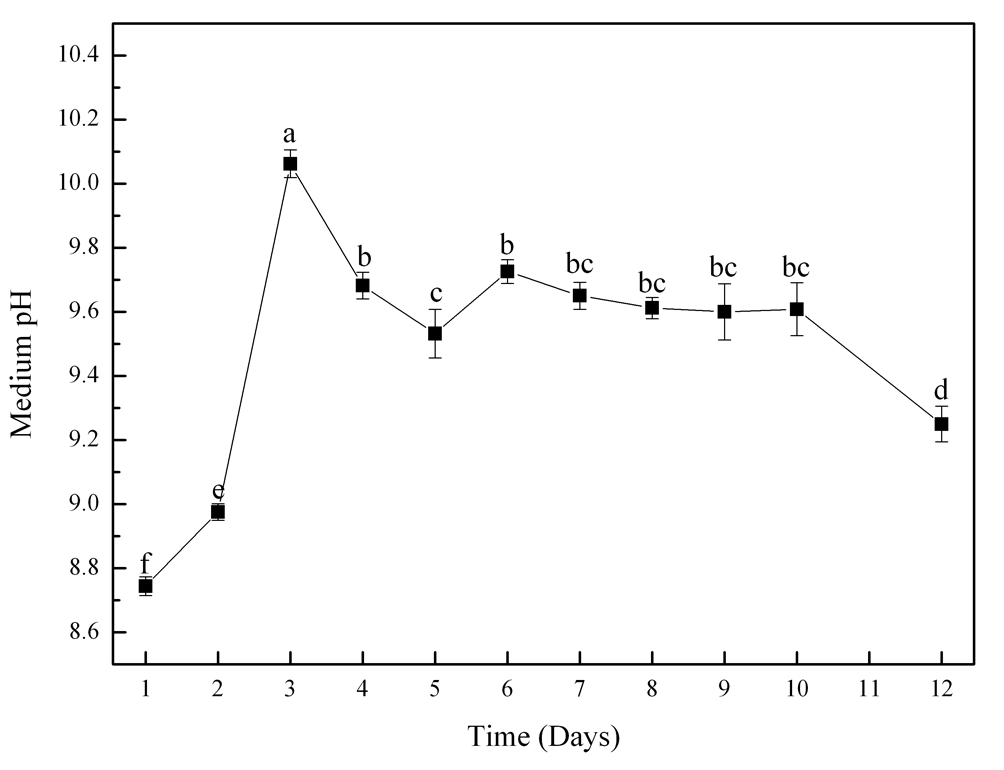
**

**Figure S1 The medium pH during the culture of *Phaeodactylum tricornutum*.** Medium pH was determined daily using a pH meter during the culture of *P. tricornutum* (for 12 days). **Each bar represents three replications. Different letters indicate statistically significant differences between treatments (Duncan’s multiple range test: P< 0.05).**


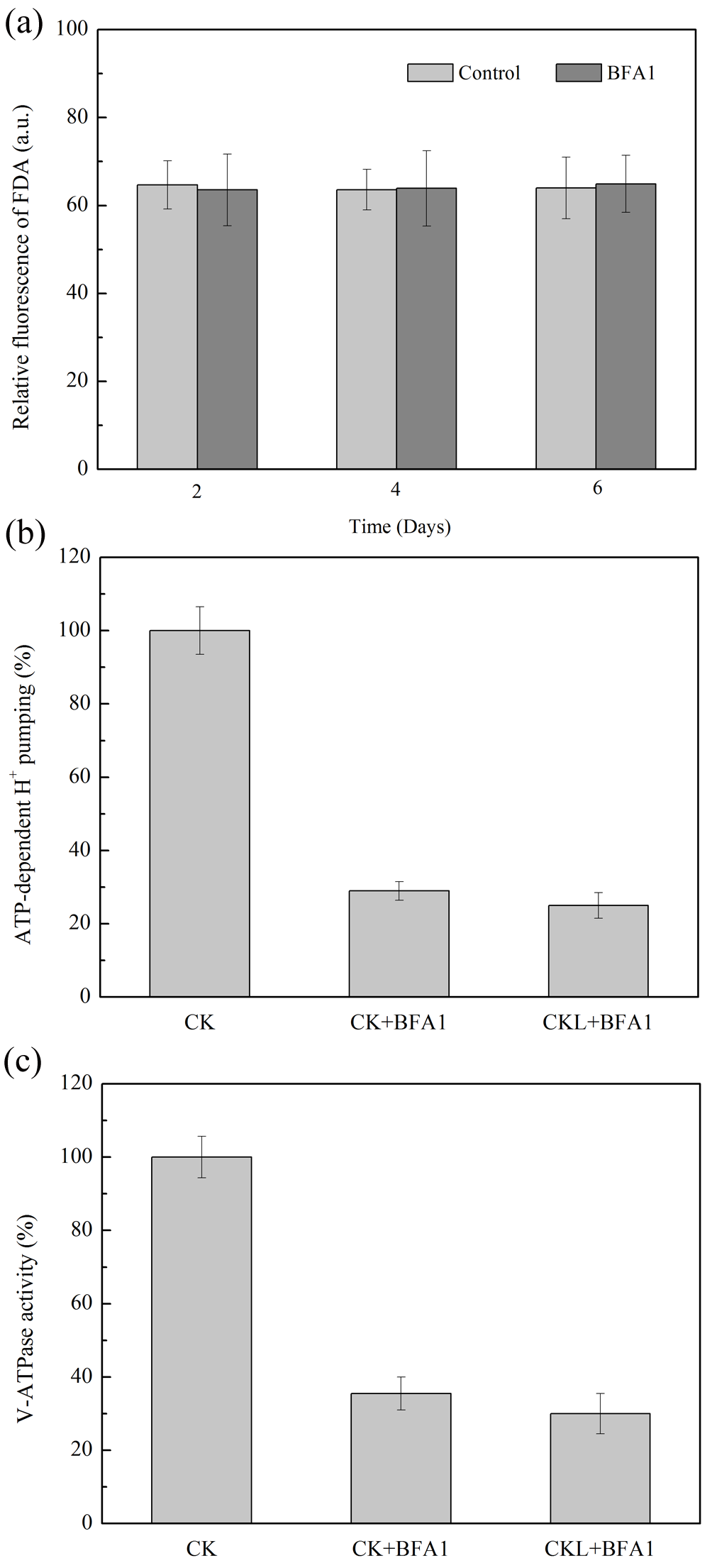


**Figure S2 The toxicity and efficiency of V-ATPase inhibitor BFA1 during the culture of *Phaeodactylum tricornutum*.** (**a**) Determination of *P. tricornutum* viability in 2, 4, 6 days after BFA1 treatment. FDA fluorescence was determined using a microtiter plate reader. Ex=488 nm; Em=530 nm. **Each bar represents three replications. (b) V-ATPase proton pumping was measured by the quenching of ACMA fluorescence. (c) V-ATPase activity was determined in BFA1 treatment and control.** The activity of V-ATPase was calculated as the difference measured in the absence or presence of 100 nM Con A. **Ten micrograms of microsomal membrane protein was applied to detect fluorescence density. Each bar represents three replications. Control (CK), no treatment; CK+BAF1, treatment with 100 nM BFA1 in the initial culture; CKL+BFA1, treatment with 100 nM BFA1 in 6 days after culture.**


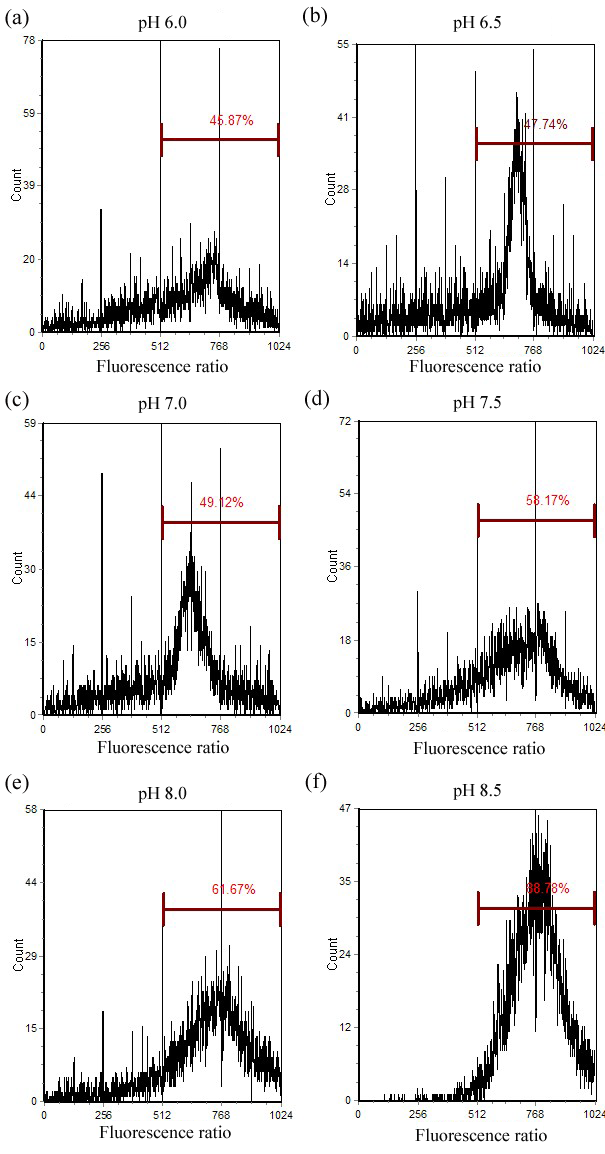


**Figure S3 Calibration of intracellular pH measurement. In situ calibration was used to determine intracellular pH. C**ell samples were centrifuged, and pellets were resuspended in high [K^+^] buffers at pH 6.0, 6.5, 7.0, 7.5, 8.0, and 8.5. Fluorescence was measured after the addition of nigericin (to equilibrate pH_i_ and pH). BCECF excitation was provided by the 488 nm line of an argon laser. When used with an Epics Elite cytometer, power as low as 15–20 mW was adequate for excitation. The resulting fluorescence was separated into high- and low-wavelength components by a 550 nm dichroic filter. These components were further narrowed by passing through 640 and 525 nm band pass filters, respectively. The ratio of 525/640 nm fluorescence was measured as reflected by intracellular pH alteration.


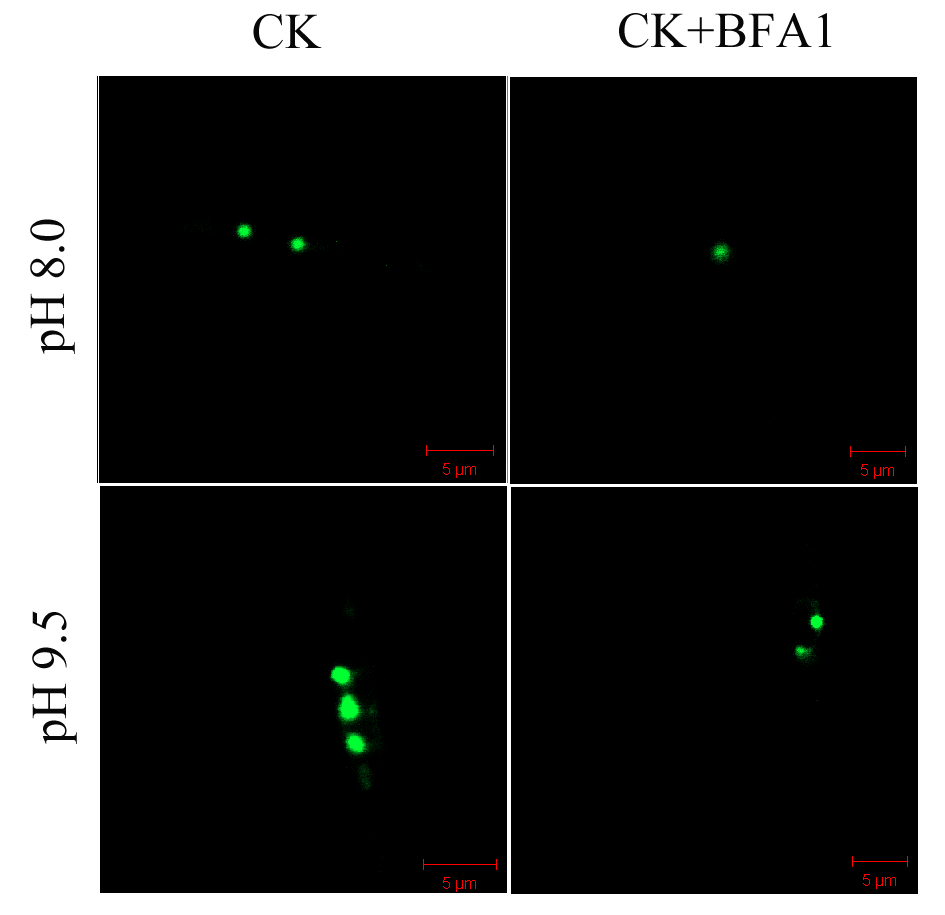


**Figure S4 Oil bodies observed by confocal microscopy combined with CHES and BFA1 treatment.** In control or CHES buffer (pH = 9.5) treated *P. tricornutum*, added 100 nM BFA1 in the initial culture or 6 days after culture, cultured with 8 days, then stained with Nile Red. Mixtures were thoroughly mixed, applied to a glass slide, covered with a coverslip after 10 min, and then observed under an LSM 510 META laser-scanning confocal microscope (Zeiss), with 543 nm excitation wavelength and 570–610 nm emission wavelength. Images were acquired randomly from at least 20 cells per sample, and typical images are presented here. Bar = 5 μm. **CK, without BFA1 treatment; CK+BAF1, treatment with 100 nM BFA1 in the initial culture.**


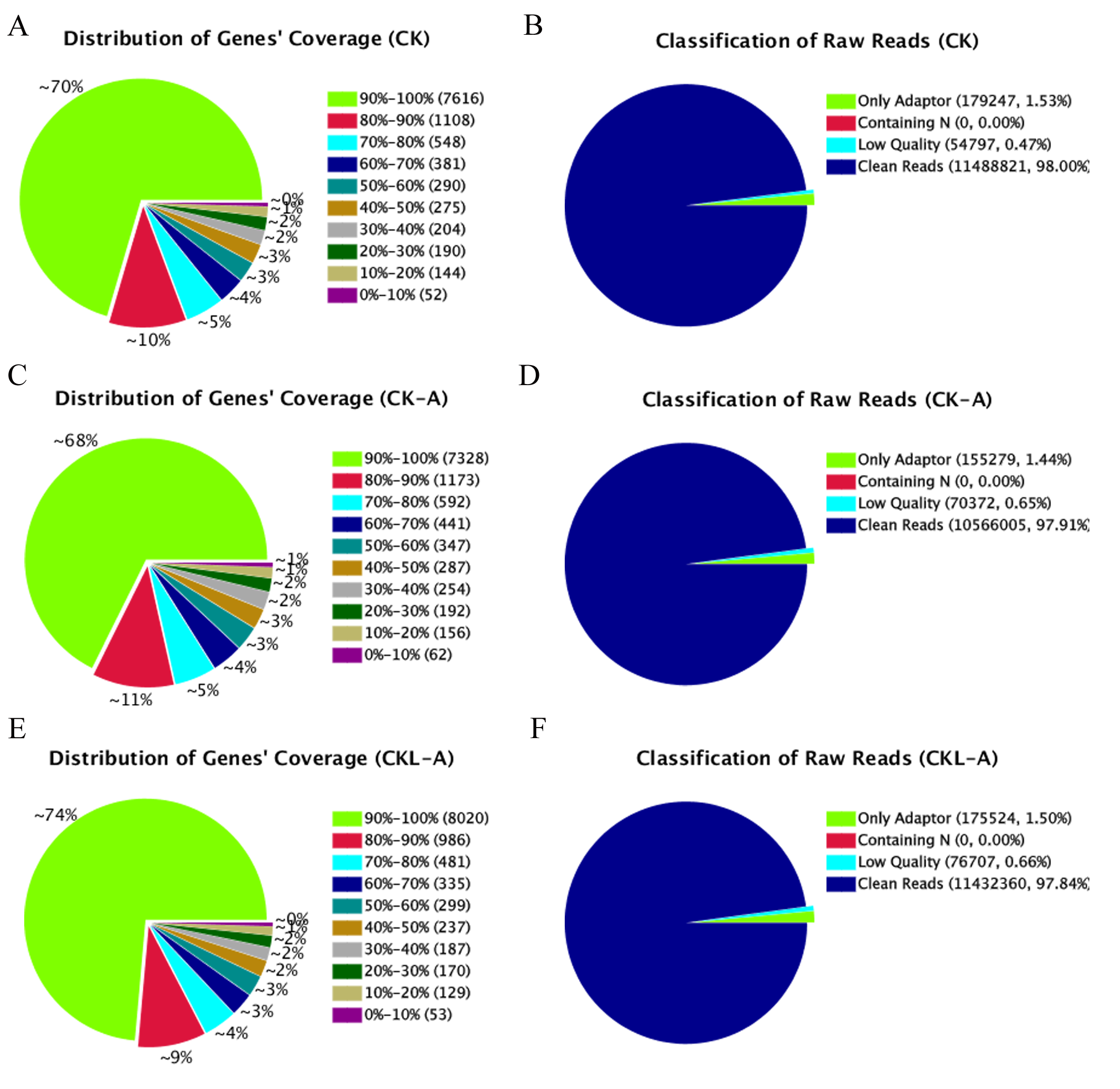


**Figure S5 Classification of raw reads and distribution of unigene size in the *Phaeodactylum tricornutum* transcriptome assembly under control and BFA1 treatment. CK, no treatment; CK-A, treatment with 100 nM BFA1 in the initial culture; CKL-A, treatment with 100 nM BFA1 in 6 days after culture.**


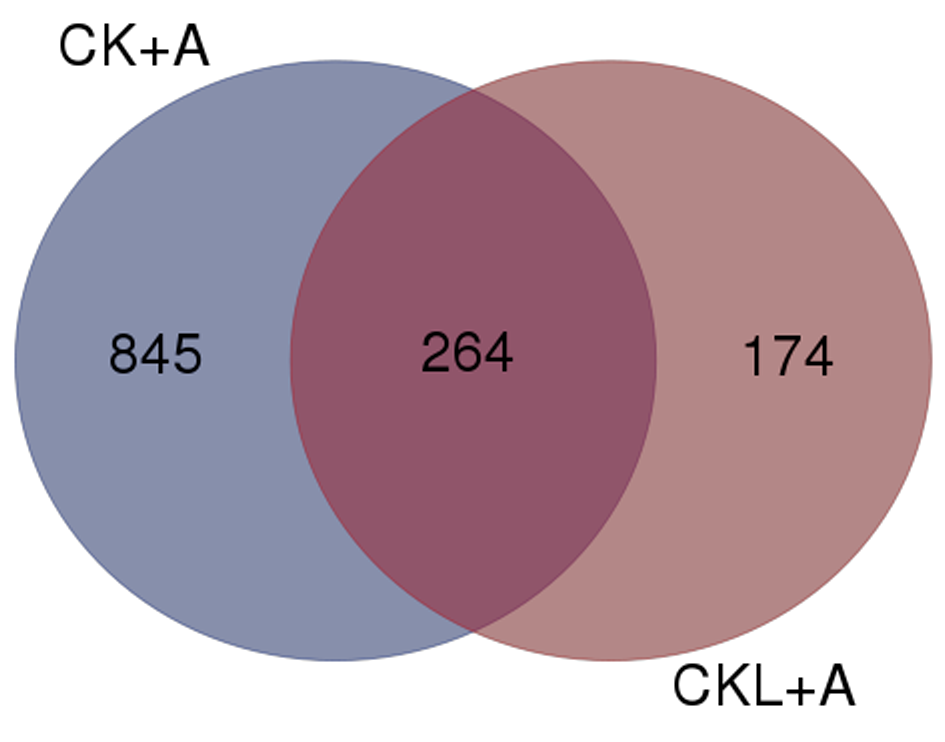


**Figure S6 Venn diagram depicting the overlap between gene DE with BFA1 treatment in the initial culture and 6 days after culture.** Differential expression with BFA1 treatment was assessed at an FDR<0.05 and fold changes>2. **CK+BAF1, treatment with 100 nM BFA1 in the initial culture; CKL+BFA1, treatment with 100 nM BFA1 in 6 days after culture.**


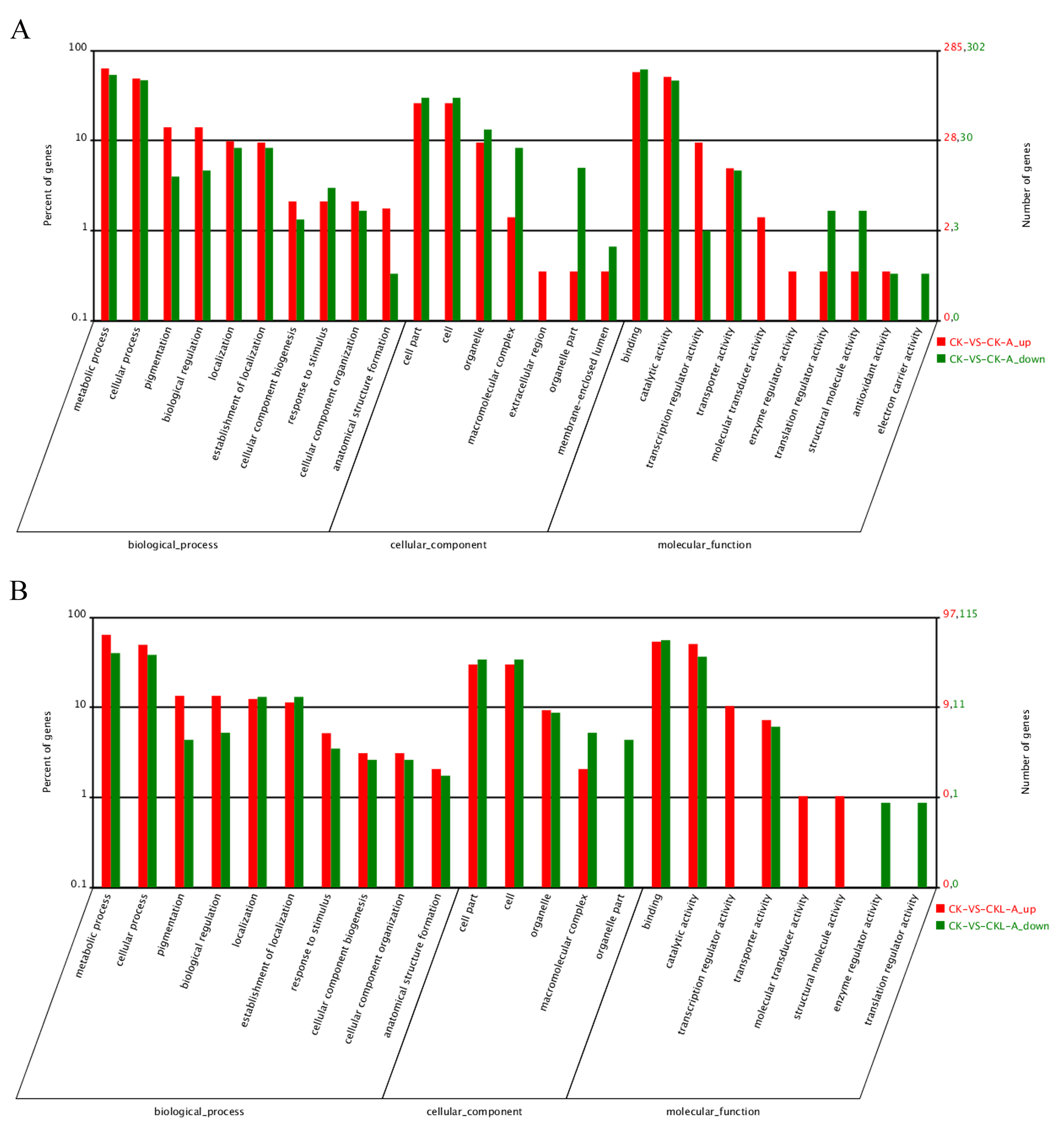


**Figure S7 Gene ontology (GO) classification of the *Phaeodactylum tricornutum* transcripts with Blast2GO program under BFA1 treatment. CK, no treatment; CK-A, treatment with 100 nM BFA1 in the initial culture; CKL-A, treatment with 100 nM BFA1 in 6 days after culture.**


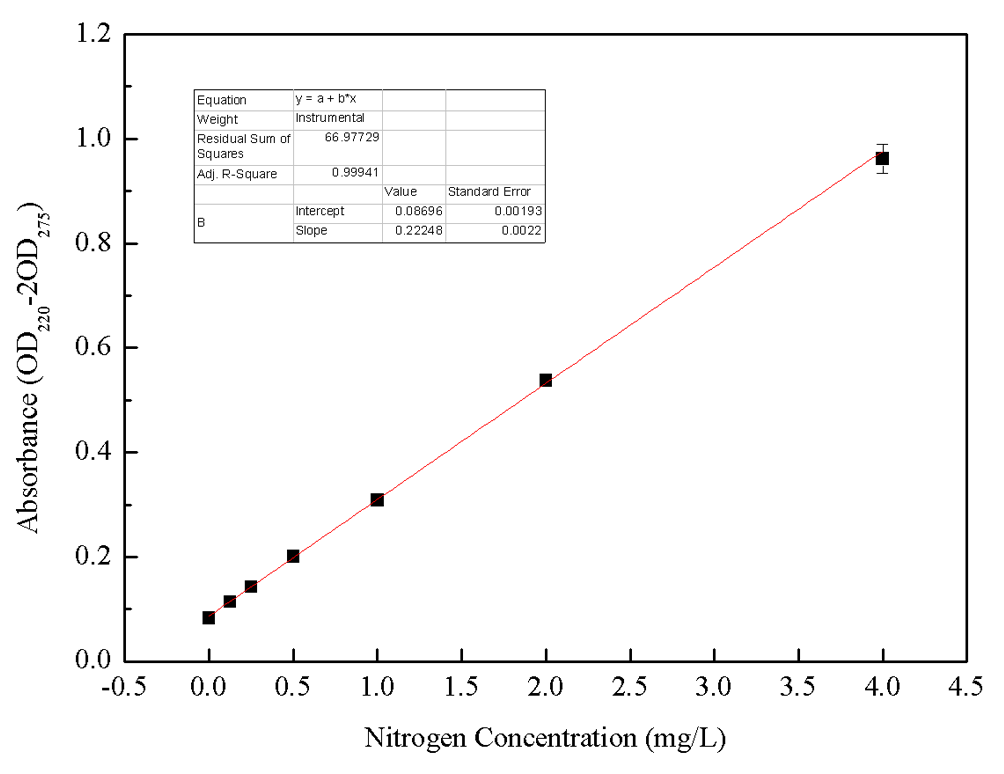


**Figure S8 Calibration of nitrogen contents.** Nitrogen content was calibrated using the colorimetric method (Cleverchem200, DeChem-Tech) in 0, 0.125, 0.25, 0.5, 1.0, 2.0, and 4.0 mg L^−1^ KNO_3_ solution.
